# Comprehensive In Silico Investigation of L-Glutamine Transporters and Metabolism in Glioblastoma

**DOI:** 10.3390/ph19030455

**Published:** 2026-03-11

**Authors:** Sachin Kumar, Chih-Yang Wang, Helena Kishore Lalwani, Juan Lorell Ngadio, Fitria Sari Wulandari, Daniel Dahlak Solomon, Hui-Pu Liu

**Affiliations:** 1 Graduate Institute of Cancer Biology and Drug Discovery, College of Medical Science and Technology, Taipei Medical University, Taipei 11031, Taiwan; sachinkumar1@shooliniuniversity.com (S.K.); chihyang@tmu.edu.tw (C.-Y.W.); helena.lalwani@student.i3l.ac.id (H.K.L.); juan.lorell@alumni.i3l.ac.id (J.L.N.); 2 PhD Program for Cancer Molecular Biology and Drug Discovery, College of Medical Science and Technology, Taipei Medical University, Taipei 11031, Taiwan; 3 Faculty of Applied Sciences and Biotechnology, Shoolini University of Biotechnology and Management Sciences, Solan 173229, India; 4 Department of Pharmacy, School of Life Sciences, Indonesia International Institute for Life Sciences, Jl Pulomas Barat Kav 88, Jakarta Timur 13210, Indonesia; 5 Department of Bioinformatics, School of Life Sciences, Indonesia International Institute for Life Sciences, Jl Pulomas Barat Kav 88, Jakarta Timur 13210, Indonesia; 6 PhD in Medical Neuroscience, College of Medical Science and Technology, Taipei Medical University, Taipei 11031, Taiwan; d620114002@tmu.edu.tw; 7 Department of General Surgery, Kaohsiung Armed Forces General Hospital, National Defense Medical University, Kaohsiung 80284, Taiwan

**Keywords:** glioblastoma, glutamine metabolism, *SLC25A13*, *SLC38A2*, ceruloplasmin, cancer metabolism

## Abstract

**Background/Objectives:** Glioblastoma (GBM) is the most aggressive primary brain tumor in adults and remains associated with poor prognosis despite multimodal therapy. Metabolic reprogramming, particularly increased dependence on glutamine, supports GBM bioenergetic, biosynthetic, and redox demands. This study aimed to systematically identify glutamine-associated metabolic regulators with prognostic relevance and biological plausibility in GBM. **Methods:** Transcriptomic data from TCGA and GTEx were analyzed using GEPIA2, with survival validation performed using the CGGA. Functional pathway enrichment, protein expression assessment, protein–protein interaction network analysis, tumor microenvironment evaluation, epigenetic profiling, and single-cell RNA sequencing validation were integrated to contextualize candidate genes. Pharmacogenomic correlation analysis and structure-based molecular docking were applied as supportive validation layers. **Results:** Ceruloplasmin (*CP*), Solute Carrier Family 25 Member 13 (*SLC25A13*), and Solute Carrier Family 38 Member 2 (*SLC38A2*) were selectively dysregulated and associated with poor clinical outcomes in GBM. CP was linked to redox regulation and stress-adaptive survival programs, *SLC25A13* to mitochondrial metabolite exchange and glutamine-coupled nucleotide biosynthesis, and *SLC38A2* to glutamine uptake, nutrient sensing, and mTORC1-MYC-associated growth signaling. **Conclusions:** *CP*, *SLC25A13*, and *SLC38A2* emerge as clinically relevant glutamine-associated metabolic regulators in GBM, linking redox regulation, mitochondrial metabolite exchange, and glutamine-driven growth signaling. These findings highlight transport- and exchange-centered metabolic nodes as potential biomarkers and candidates for future metabolic targeting in GBM.

## 1. Introduction

Glioblastoma (GBM) represents the most aggressive form of primary brain cancer in adults and remains largely incurable despite decades of therapeutic advances [1,2]. The limited clinical efficacy of current standard-of-care treatments reflects not only the genetic heterogeneity of GBM but also its extraordinary capacity for metabolic adaptation. Tumor cells within the brain must survive fluctuating nutrient availability, hypoxia, and oxidative stress, requiring continuous rewiring of core metabolic programs. Understanding how GBM sustains these metabolic states is therefore central to identifying biologically meaningful vulnerabilities [3,4].

Amino acid metabolism has emerged as a critical determinant of tumor fitness, with glutamine occupying a central role in cancer cell survival [5]. Glutamine supports cellular proliferation by providing carbon for mitochondrial metabolism, nitrogen for nucleotide and amino acid biosynthesis, and reducing equivalents for redox homeostasis. While glutamine is dispensable for most normal cells, many cancers exhibit a heightened dependence on extracellular glutamine, reflecting a shift toward anabolic metabolism [6]. In GBM, this dependency is particularly pronounced due to the high energetic demands imposed by rapid proliferation and adaptation to the metabolically constrained brain microenvironment. Rather than relying on a single metabolic reaction, glutamine utilization in cancer is governed by an integrated network of transporters, mitochondrial carriers, and metabolic enzymes that collectively regulate glutamine uptake, intracellular trafficking, and metabolic flux distribution. Plasma membrane transporters facilitate glutamine entry into tumor cells, while mitochondrial carriers enable efficient coupling of glutamine-derived metabolites to the tricarboxylic acid cycle, nucleotide synthesis, and redox balance. This coordinated regulation allows tumor cells to dynamically allocate glutamine toward biosynthetic or bioenergetic pathways in response to environmental stress. In GBM, glutamine metabolism is further shaped by oncogenic signaling pathways and transcriptional programs that reinforce metabolic dependency. However, emerging evidence suggests that glutamine-associated metabolic reprogramming is not uniform across all related genes. Instead, GBM appears to selectively amplify specific transport and exchange nodes that support tumor growth while suppressing others that may be incompatible with the malignant state. Although well-characterized metabolic drivers such as IDH mutations have provided important insights into glioma biology, they do not fully explain the broader metabolic landscape of GBM, nor do they capture transport-centric mechanisms that regulate glutamine availability and utilization [6,7,8].

Despite the recognized importance of glutamine metabolism in GBM, a systematic evaluation of glutamine-associated genes integrating transcriptional deregulation, clinical relevance, pathway context, epigenetic regulation, tumor cell specificity, and functional perturbability remains limited. In particular, glutamine transporters and mitochondrial carriers have been relatively underexplored as biomarkers and potential metabolic control points, even though they occupy key positions at the interface between nutrient availability and intracellular metabolic programs [7]. In this study, we conducted an integrative in silico analysis to comprehensively characterize glutamine-associated metabolic regulators in GBM. Using transcriptomic data from The Cancer Genome Atlas (TCGA) and the Genotype-Tissue Expression (GTEx), we identified genes exhibiting tumor-specific expression patterns, followed by survival analysis using Chinese Glioma Genome Atlas (CGGA) to determine clinical relevance. Functional pathway enrichment, protein-level validation, epigenetic profiling, tumor microenvironment analysis, and Single-cell RNA sequencing (scRNA-seq) were employed to contextualize candidate genes within GBM biology. Finally, pharmacogenomic correlation analysis and structural modeling were incorporated as supportive validation layers to assess the feasibility of perturbing these metabolic nodes. Through this multi-dimensional framework, we aim to identify glutamine-associated regulators that reflect dominant metabolic states in GBM and may serve as biologically meaningful biomarkers and candidates for future metabolic targeting strategies. 

## 2. Results

### 2.1. Study Design and Transcriptomic Analysis of Glutamine-Associated Genes in Glioblastoma

**Figure 1:**
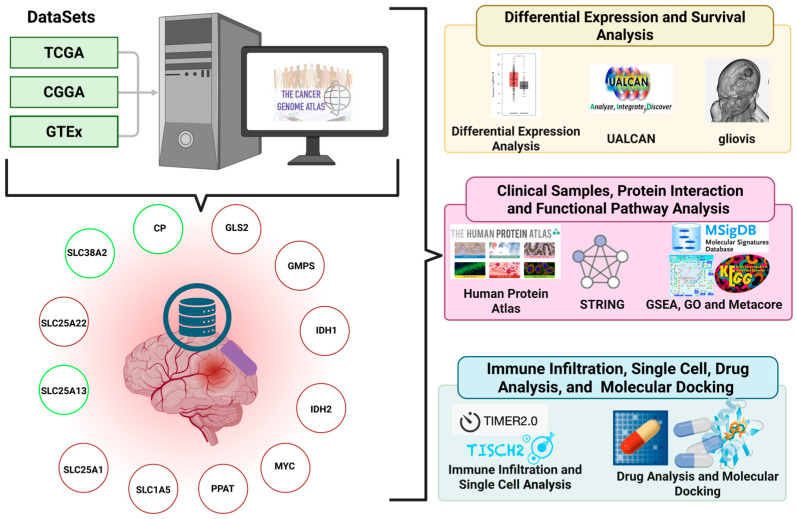
**Overview of the integrative analytical workflow used to identify glutamine-associated metabolic regulators in GBM.** Publicly available transcriptomic datasets from TCGA, CGGA, and GTEx were integrated to perform differential expression and survival analyses using platforms including GEPIA2, UALCAN, and Gliovis. Candidate glutamine-associated genes were prioritized based on expression patterns and prognostic relevance. Protein-level validation and functional context were assessed using the Human Protein Atlas (HPA) and protein–protein interaction networks constructed via STRING. Pathway enrichment analyses were conducted using GSEA, Gene Ontology (GO), KEGG, and MetaCore to delineate biological processes linked to the selected genes. Immune infiltration analysis, scRNA-seq validation, pharmacogenomic screening, and structure-based molecular docking were subsequently performed to evaluate tumor microenvironment associations and therapeutic relevance, forming a comprehensive in silico framework for identifying metabolic vulnerabilities in GBM.

To systematically investigate glutamine-associated metabolic alterations in GBM, we adopted an integrated in silico study design combining transcriptomic profiling with biological and clinical prioritization. Publicly available RNA-sequencing data from TCGA and GTEx project were utilized to capture tumor-specific transcriptional changes while minimizing confounding effects from normal brain physiology. This strategy enabled the identification of metabolic genes that are selectively deregulated in GBM. Based on an extensive literature-guided framework focusing on glutamine transport, mitochondrial metabolism, and nucleotide biosynthesis, a focused list of candidate genes was defined. This list included *CP*, *GLS2*, *GMPS*, *IDH1*, *IDH2*, *MYC*, *PPAT*, *SLC1A5*, *SLC25A1*, *SLC25A13*, *SLC25A22*, and *SLC38A2*, representing key nodes linking glutamine utilization to biosynthetic and proliferative pathways (Figure 1).

**Figure 2:**
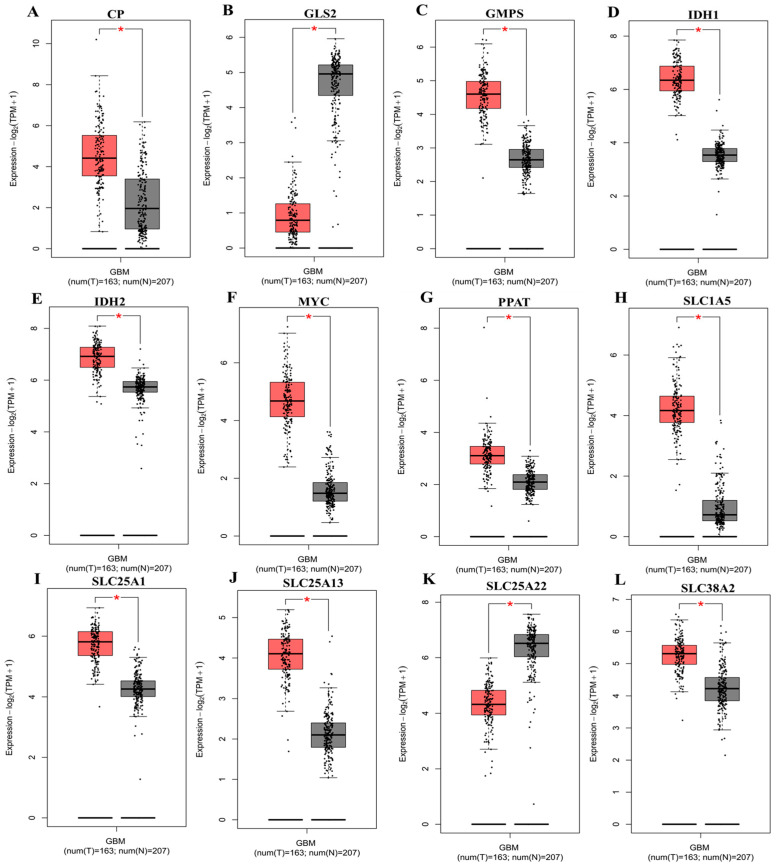
**Differential expression of glutamine-associated metabolic and transporter genes in GBM.** Box plots depicting mRNA expression levels of selected glutamine-related genes in GBM tumor tissues compared with normal brain tissues, analyzed using GEPIA2 based on TCGA and GTEx datasets. The genes shown include (**A**) *CP*, (**B**) *GLS2*, (**C**) *GMPS*, (**D**) *IDH1*, (**E**) *IDH2*, (**F**) *MYC*, (**G**) *PPAT*, (**H**) *SLC1A5*, (**I**) *SLC25A1*, (**J**) *SLC25A13*, (**K**) *SLC25A22*, and (**L**) *SLC38A2*. Expression values are presented as log_2_ (TPM + 1). Red boxes represent GBM tumor samples, while grey boxes indicate normal brain samples. Each dot corresponds to an individual sample, with sample numbers shown below each panel (tumor, n = 163; normal, n = 207). Statistical significance between tumor and normal tissues was determined by the GEPIA2 default differential expression pipeline; *p* < 0.05 is indicated by an asterisk.

To further evaluate transcriptional deregulation of these genes, differential expression analysis was performed using the GEPIA2 platform. Gene expression levels were compared between GBM tumor samples and normal brain tissues derived from TCGA and GTEx datasets. Several genes demonstrated significant tumor-associated upregulation, including *CP*, *GMPS*, *MYC*, *PPAT*, *SLC1A5*, *SLC25A1*, *SLC25A13*, and *SLC38A2*, indicating activation of glutamine transport, mitochondrial metabolite exchange, and nucleotide biosynthesis programs in malignant cells (Figure 2A–L). In contrast, *GLS2*, and *SLC25A22* displayed relatively higher expression in normal brain tissue, highlighting that GBM does not globally upregulate all components of glutamine metabolism but instead exhibits selective pathway rewiring.

**Figure 3:**
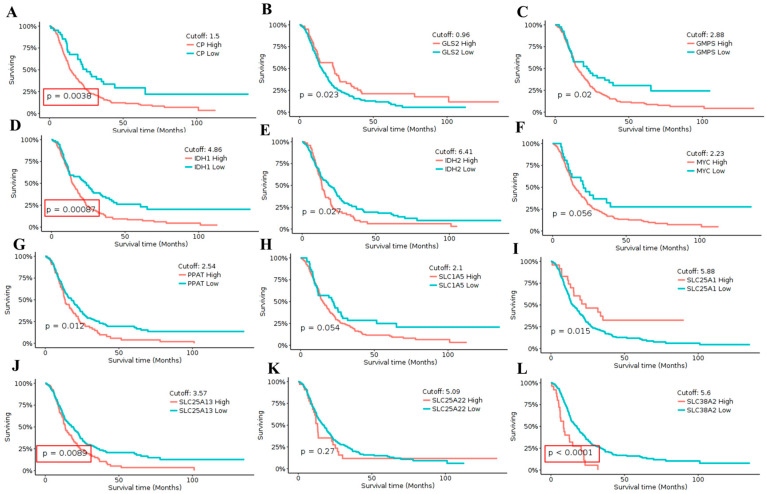
**Survival impact of glutamine-associated genes in GBM.** Kaplan–Meier overall survival curves illustrating the prognostic relevance of selected glutamine transporters and metabolic regulators in glioma patients, generated using the GlioVis platform.(CGGA) Panels show survival stratification for (**A**) *CP*, (**B**) *GLS2*, (**C**) *GMPS*, (**D**) *IDH1*, (**E**) *IDH2*, (**F**) *MYC*, (**G**) *PPAT*, (**H**) *SLC1A5*, (**I**) *SLC25A1*, (**J**) *SLC25A13*, (**K**) *SLC25A22*, and (**L**) *SLC38A2*. Patients were divided into high- and low-expression groups based on gene-specific optimal cutoff values displayed in each panel. Red curves indicate high expression, whereas blue curves indicate low expression. Statistical significance was assessed using the log-rank test, with corresponding *p*-values shown on the plots. Elevated expression of *CP*, *GMPS*, *IDH1*, *IDH2*, *PPAT*, *SLC25A1*, *SLC25A13*, and *SLC38A2* was associated with significantly reduced overall survival, while other genes exhibited weaker or non-significant survival associations.

To determine the clinical relevance of these transcriptional alterations, survival analysis was subsequently performed using GlioVis. High expression of *CP* was significantly associated with poorer overall survival (*p* = 0.0038), indicating a strong adverse prognostic impact (Figure 3A). Similarly, elevated expression of *GMPS* (*p* = 0.02), *IDH1* (*p* = 0.00087), *PPAT* (*p* = 0.012), *SLC25A1* (*p* = 0.015), *SLC25A13* (*p* = 0.0089), and *SLC38A2* (*p* < 0.0001) was associated with significantly reduced survival, supporting their potential involvement in aggressive disease behavior (Figure 3C,D,G,I,J,L). In contrast, *MYC* (*p* = 0.056), *SLC1A5* (*p* = 0.054), and *SLC25A22* (*p* = 0.27) did not reach statistical significance, indicating limited prognostic contribution in this cohort (Figure 3F,H,K). Among the genes showing the strongest and most consistent survival associations, *CP*, *IDH1*, *SLC25A13*, and *SLC38A2* emerged as the most statistically significant candidates based on both expression and outcome analyses (Figure 2A–L and Figure 3A–L). Although *IDH1* demonstrated a highly significant survival association, it was not pursued further due to its well-established and extensively characterized role as a canonical glioma biomarker, largely driven by mutation-dependent mechanisms rather than transcriptional regulation (Figure 3D).

Based on the convergence of tumor-specific upregulation and robust adverse survival associations, *CP*, *SLC25A13*, and *SLC38A2* were selected for further study. This selection strategy prioritized relatively underexplored metabolic and transport-associated nodes within glutamine metabolism that may represent clinically relevant and targetable vulnerabilities in GBM.

### 2.2. Integrated Clinical, Molecular, and Network Characterization of CP, SLC25A13, and SLC38A2 in GBM

**Figure 4:**
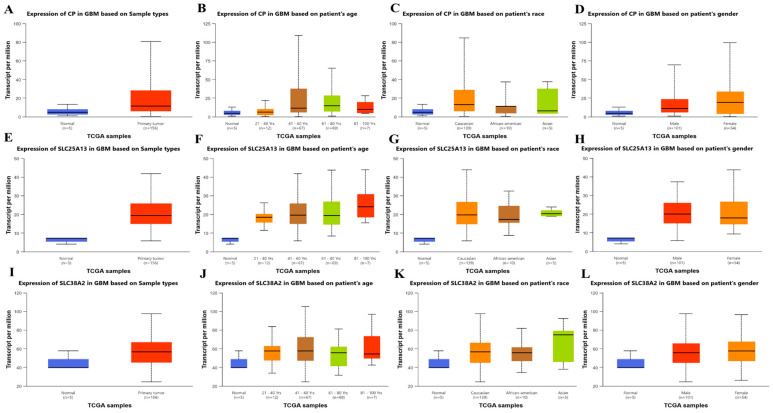
**Association of CP, SLC25A13, and SLC38A2 expression with clinicopathological features in GBM.** Box plots showing the expression levels of *CP*, *SLC25A13*, and *SLC38A2* in GBM) samples stratified by clinical and demographic variables using TCGA-derived data. Panels (**A**–**D**) illustrate *CP* expression according to sample type (normal vs. primary tumor), patient age group, race, and gender. Panels (**E**–**H**) show corresponding stratifications for *SLC25A13*, and panels (**I**–**L**) depict *SLC38A2* expression across the same clinical categories. Gene expression is presented as transcripts per million. Sample numbers for each subgroup are indicated below the respective plots. Across all three genes, higher expression levels are observed in primary GBM tumors compared with normal brain tissue, with variable distributions across age, race, and gender subgroups.

Following transcriptomic prioritization, clinical relevance, protein-level expression, and functional network integration of *CP*, *SLC25A13*, and *SLC38A2* were systematically examined to establish their biological roles in GBM. Expression stratification across clinicopathological variables demonstrated that all three genes exhibit consistently higher transcript levels in primary GBM tumors compared with normal brain tissue, with variable distributions across patient age, race, and gender categories (Figure 4A–L). The predominance of tumor-associated expression across all strata indicates that transcriptional activation of these genes is driven primarily by malignant transformation rather than demographic factors. From a biological perspective, this pattern is consistent with GBM-associated metabolic stress. *CP* is closely linked to iron metabolism and redox homeostasis, processes that are critical in hypoxic and oxidative tumor microenvironments. *SLC25A13*, a mitochondrial aspartate-glutamate carrier, plays a central role in coupling mitochondrial metabolism with cytosolic nucleotide biosynthesis by facilitating nitrogen and carbon flux. *SLC38A2*, a sodium-coupled neutral amino acid transporter, supports adaptive amino acid uptake under nutrient-limited conditions, a hallmark of highly proliferative GBM cells.

**Figure 5:**
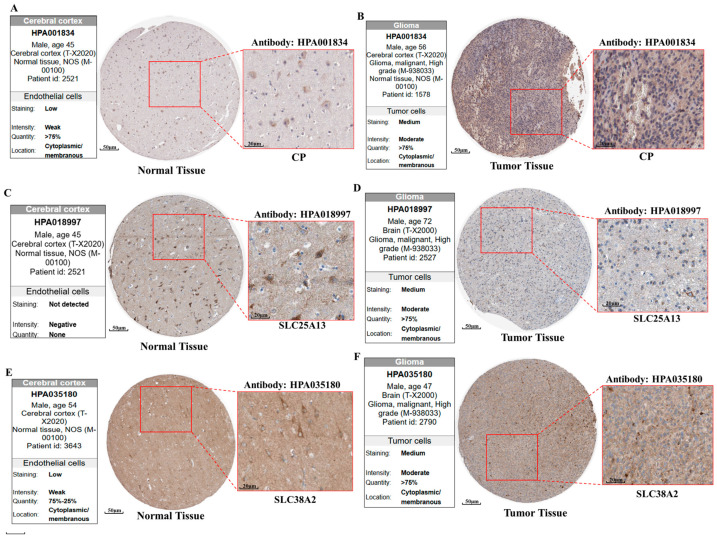
**Protein expression of *CP*, *SLC25A13*, and *SLC38A2* in GBM and normal brain tissue.** (**A**) CP expression in normal cerebral cortex showing weak staining with limited cellular positivity (antibody: HPA001834). (**B**) *CP* expression in GBM tissue demonstrating increased staining intensity and widespread cytoplasmic/membranous localization in tumor cells (antibody: HPA001834). (**C**) *SLC25A13* expression in normal cerebral cortex with no detectable staining (antibody: HPA018997). (**D**) *SLC25A13* expression in GBM tissue showing moderate staining intensity and broad tumor cell positivity with cytoplasmic/membranous localization (antibody: HPA018997). (**E**) *SLC38A2* expression in normal cerebral cortex exhibiting weak to low staining levels (antibody: HPA035180). (**F**) *SLC38A2* expression in GBM tissue displaying increased protein expression with moderate intensity and widespread cellular distribution (antibody: HPA035180). For each panel, whole-section images are shown alongside higher-magnification views highlighting cellular staining patterns. Compared with normal brain tissue, GBM samples exhibit elevated protein expression of *CP*, *SLC25A13*, and *SLC38A2*, supporting tumor-associated activation of glutamine-related metabolic pathways.

Protein-level validation further supported the functional relevance of these transcriptional changes. Immunohistochemical analysis revealed weak or absent staining of *CP*, *SLC25A13*, and *SLC38A2* in normal cerebral cortex, whereas GBM tissues displayed increased staining intensity and broader cellular positivity (Figure 5A–F). Predominantly cytoplasmic and membranous localization patterns were observed, consistent with the established transport and metabolic functions of these proteins. The concordance between mRNA upregulation and protein expression indicates active engagement of these pathways at the functional level rather than passive transcriptional dysregulation.

**Figure 6:**
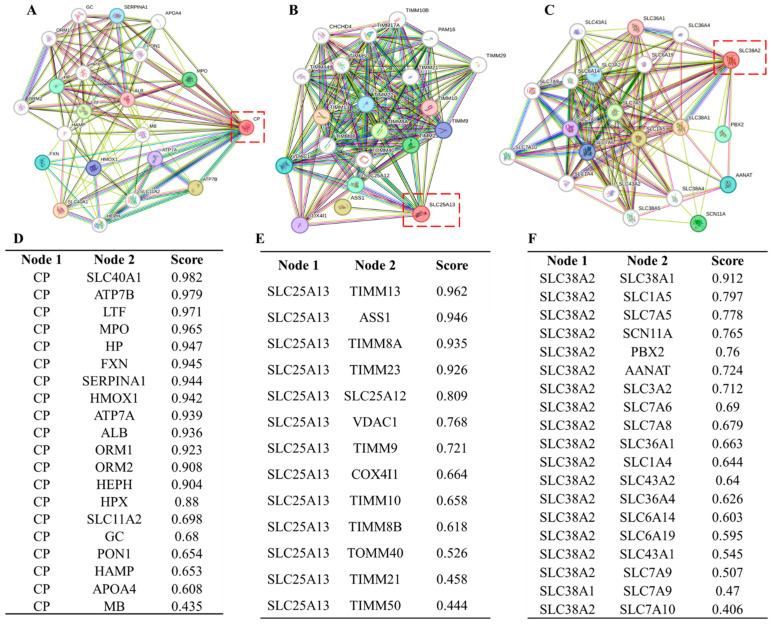
**Protein–protein interaction networks of *CP*, *SLC25A13*, and *SLC38A2* in GBM.** (**A**–**C**) Network visualizations centered on *CP* (**A**), *SLC25A13* (**B**), and *SLC38A2* (**C**), with the target genes highlighted by dashed red boxes. Nodes represent interacting proteins, and edges indicate known or predicted interactions based on curated databases, experimental evidence, and co-expression. (**D**–**F**) Tables listing the top interacting partners for *CP* (**D**), *SLC25A13* (**E**), and *SLC38A2* (**F**) ranked by interaction confidence score. These networks reveal that *CP* is primarily connected to iron homeostasis and oxidative stress–related proteins, *SLC25A13* interacts predominantly with mitochondrial transport and metabolic regulators, and *SLC38A2* shows extensive connectivity with amino acid transporters and metabolic signaling components.

To further contextualize their biological roles, protein–protein interaction network analysis was performed. *CP* demonstrated strong connectivity with proteins involved in iron handling, oxidative stress response, and inflammatory regulation, suggesting integration into redox-sensitive metabolic networks that support tumor survival under oxidative pressure (Figure 6A,D). *SLC25A13* showed extensive interactions with mitochondrial transport machinery and metabolic enzymes, highlighting its role as a hub coordinating mitochondrial–cytosolic metabolite exchange and biosynthetic flux (Figure 6B,E). *SLC38A2* exhibited broad connectivity with amino acid transporters and metabolic signaling components, consistent with its function in maintaining amino acid availability and metabolic plasticity in GBM cells (Figure 6C,F).

Collectively, the convergence of clinicopathological expression patterns, protein-level activation, and central positioning within metabolic interaction networks underscores the biological relevance of *CP*, *SLC25A13*, and *SLC38A2* in GBM (Figure 4A–L, Figure 5A–F and Figure 6A–F). These integrated features support their selection as key glutamine-associated metabolic nodes for subsequent functional enrichment, prognostic refinement, and therapeutic exploration. 

### 2.3. Integrated Functional and Pathway-Level Characterization of CP, SLC25A13, and SLC38A2 in GBM

To elucidate the biological programs associated with *CP*, *SLC25A13*, and *SLC38A2*, functional enrichment analyses were performed to map their correlated gene sets to Gene Ontology (GO), KEGG pathways, curated signaling maps, and Hallmark gene sets. Collectively, these analyses reveal a convergent activation of metabolic, biosynthetic, stress-response, and proliferative programs that are central to GBM progression.

**Figure 7:**
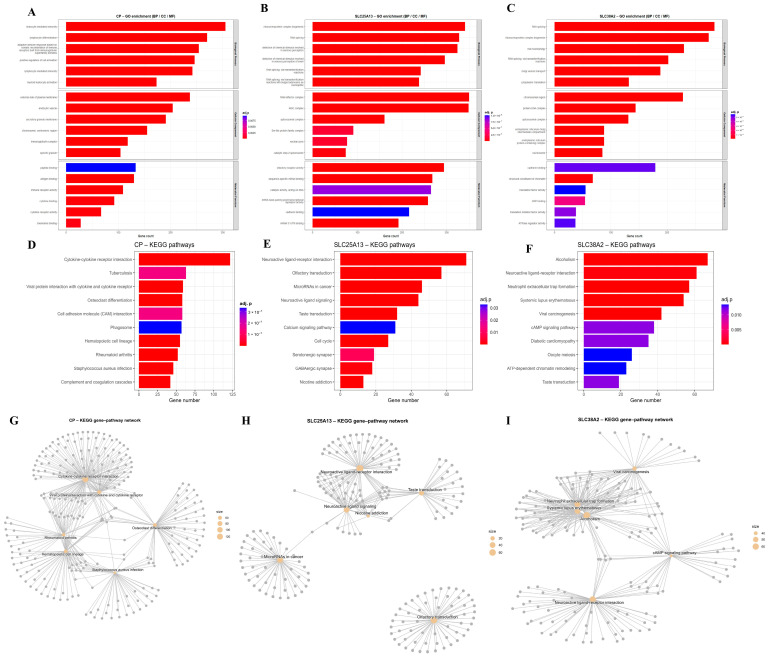
**Functional enrichment and network analysis of *CP-*, *SLC25A13-*, and *SLC38A2*-associated genes in GBM.** (**A**–**C**) GO enrichment analysis for *CP* (**A**), *SLC25A13* (**B**), and *SLC38A2* (**C**). Enriched GO terms are shown across Biological Process (BP), Cellular Component (CC), and Molecular Function (MF) categories. Bar length represents gene count, and color intensity indicates adjusted *p*-values, highlighting pathways related to immune regulation, metabolic processes, and cellular signaling. (**D**–**F**) KEGG pathway enrichment analysis for *CP* (**D**), *SLC25A13* (**E**), and *SLC38A2* (**F**). Bars represent the number of genes involved in each pathway, with color coding reflecting statistical significance. Enriched pathways include cytokine–cytokine receptor interaction, neuroactive ligand–receptor interaction, oxidative and inflammatory pathways, and metabolism-related signaling cascades. (**G**–**I**) Network visualization of enriched GO and KEGG pathways for *CP* (**G**), *SLC25A13* (**H**), and *SLC38A2* (**I**). Nodes represent enriched functional terms, and edges indicate shared gene membership between pathways. Network topology highlights functional clustering and interconnected biological processes associated with each gene.

GO enrichment demonstrated that genes positively correlated with *CP*, *SLC25A13*, and *SLC38A2* are predominantly involved in biological processes related to metabolic regulation, cellular stress adaptation, biosynthetic activity, and cell cycle control, indicating that these genes are embedded within high-demand cellular states characteristic of malignant growth (Figure 7A–C). At the pathway level, KEGG analysis revealed enrichment of metabolic and signaling pathways linked to nucleotide metabolism, amino acid transport, oxidative phosphorylation, DNA damage response, and cancer-associated signaling cascades, underscoring the integration of glutamine-related transport and metabolism with proliferative control mechanisms (Figure 7D–F). Network-based visualization of enriched gene sets further highlighted the extensive connectivity of *CP*-, *SLC25A13*-, and *SLC38A2*-associated genes, forming densely interconnected modules rather than isolated functional clusters (Figure 7G–I). This network topology supports the concept that these genes act as central coordinators within broader metabolic and regulatory systems, rather than functioning through single linear pathways. *SLC25A13*-centered networks emphasize mitochondrial transport and metabolic coupling, while *SLC38A2*-centered networks are enriched for amino acid transporters and nutrient-sensing components, reflecting adaptive responses to metabolic stress.

**Figure 8:**
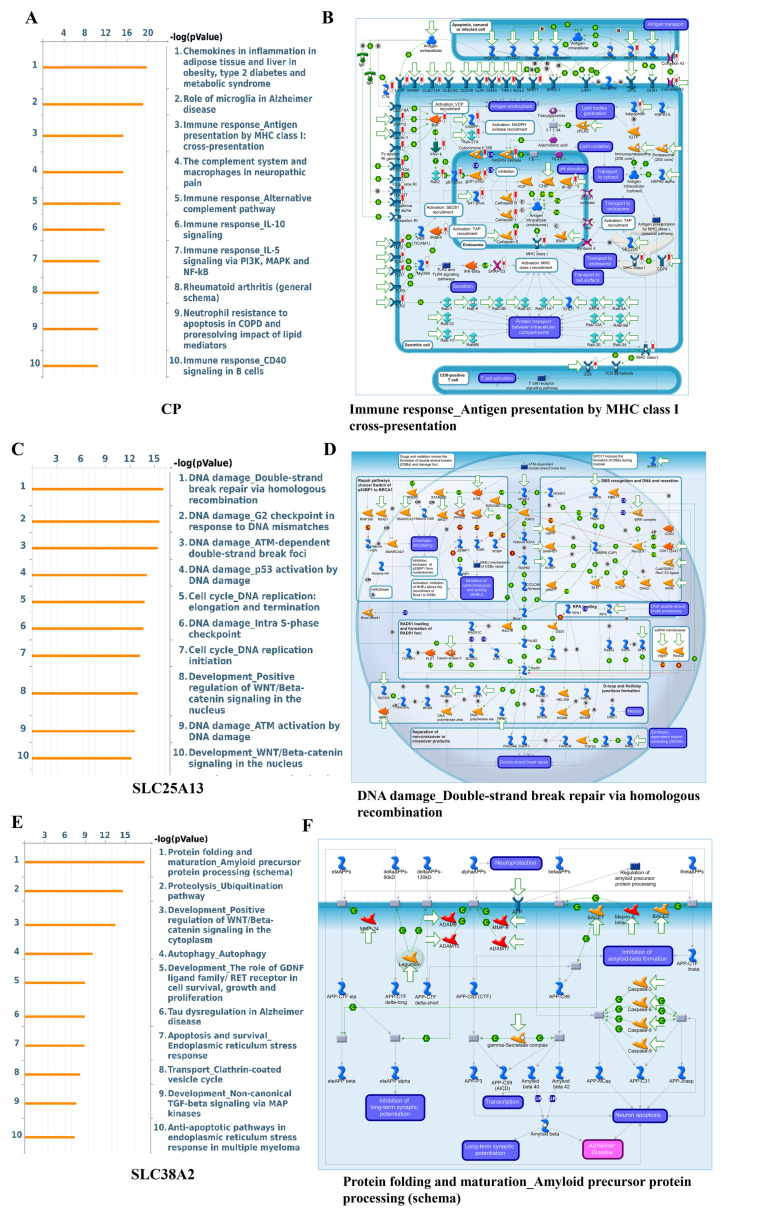
**MetaCore pathway enrichment analysis of *CP-*, *SLC25A13*-, and *SLC38A2*-associated gene networks in GBM.** (**A**,**C**,**E**) Bar plots showing the top enriched MetaCore pathway maps ranked by −log(*p*-value) for *CP*- (**A**), *SLC25A13*- (**C**), and *SLC38A2*-associated (**E**) gene sets. Enriched pathways include immune and inflammatory signaling, DNA damage response and cell cycle regulation, and stress-response–related processes. (**B**,**D**,**F**) Representative MetaCore pathway maps corresponding to the top-ranked functional categories for *CP* (**B**), *SLC25A13* (**D**), and *SLC38A2* (**F**), highlighting interconnected signaling cascades and regulatory nodes.

To refine pathway-level interpretation, curated signaling maps were examined using MetaCore analysis. *CP*-associated gene sets showed enrichment of inflammatory and immune-modulatory signaling, including chemokine- and cytokine-driven pathways, consistent with *CP*’s established role in redox balance and iron-associated inflammatory responses within the tumor microenvironment (Figure 8A). In contrast, *SLC25A13*-correlated genes were strongly associated with DNA damage response, cell cycle checkpoint regulation, and replication stress pathways, including ATM/ATR signaling and S-phase control, indicating a tight coupling between mitochondrial metabolite transport and genome maintenance during rapid cell division (Figure 8B). *SLC38A2*-associated networks were enriched for protein folding, stress-response signaling, and growth factor–driven pathways, reflecting its role in sustaining amino acid availability under conditions of nutrient limitation and oncogenic stress (Figure 8C).

**Figure 9:**
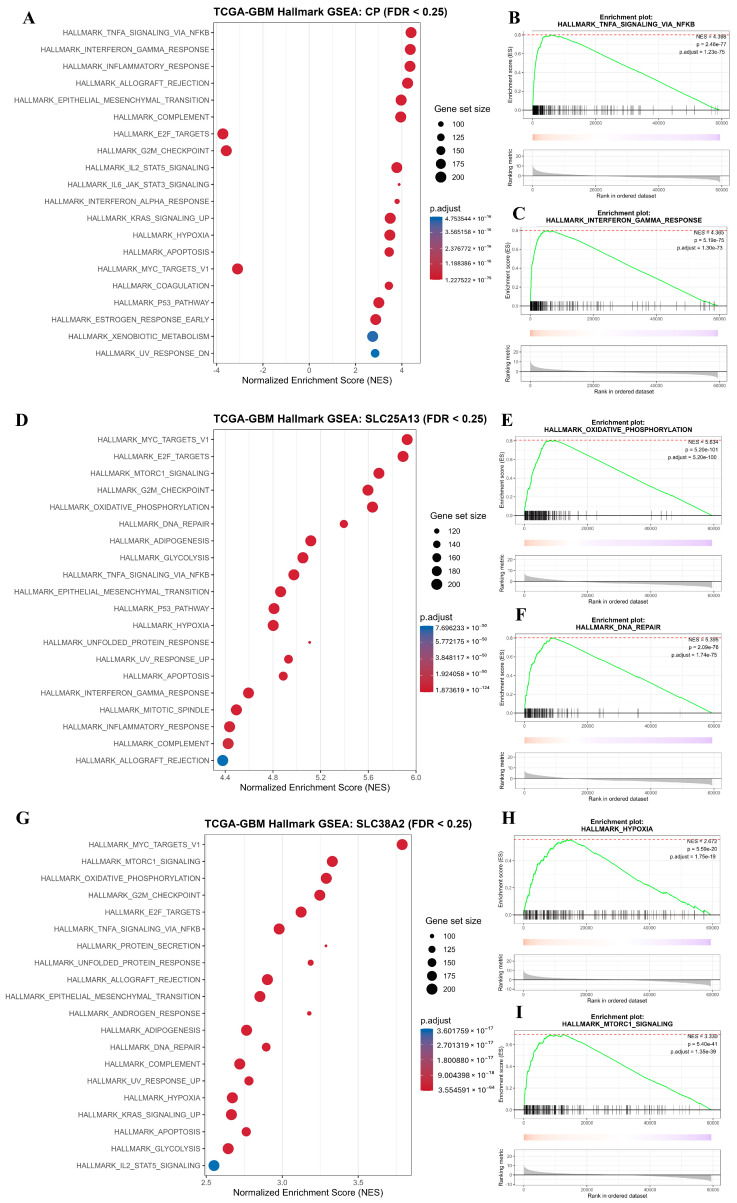
**Hallmark gene set enrichment analysis of *CP*, *SLC25A13*, and *SLC38A2* in GBM using TCGA-GBM transcriptomic data.** (**A**) Bubble plot summarizing significantly enriched Hallmark pathways associated with CP expression (FDR < 0.25), displayed according to normalized enrichment score (NES). Bubble size represents gene set size, and color indicates adjusted *p*-value. (**B**,**C**) Representative GSEA enrichment plots for HALLMARK_TNFA_SIGNALING_VIA_NFKB (**B**) and HALLMARK_INTERFERON_GAMMA_RESPONSE (**C**), highlighting immune and inflammatory signaling associated with *CP*. (**D**) Bubble plot showing Hallmark pathways significantly enriched in association with *SLC25A13* expression (FDR < 0.25). Enriched pathways include metabolic regulation, cell cycle control, oxidative phosphorylation, and DNA repair. (**E**,**F**) Representative enrichment plots for HALLMARK_OXIDATIVE_PHOSPHORYLATION (**E**) and HALLMARK_DNA_REPAIR (**F**) linked to *SLC25A13* expression. (**G**) Bubble plot illustrating significantly enriched Hallmark pathways associated with *SLC38A2* expression (FDR < 0.25), including MYC targets, mTORC1 signaling, hypoxia, and metabolic pathways. (**H**,**I**) Representative enrichment plots for HALLMARK_HYPOXIA (**H**) and HALLMARK_MTORC1_SIGNALING (**I**) associated with *SLC38A2* expression.

Consistent with these findings, Hallmark gene set enrichment analysis revealed coherent transcriptional programs associated with high expression of each gene. *CP* expression correlated with enrichment of inflammatory signaling pathways, including TNFα signaling via NF-κB, interferon responses, epithelial–mesenchymal transition, and cell cycle–associated gene sets, indicating a link between metabolic stress, inflammation, and invasive tumor phenotypes (Figure 9A–C). Elevated *SLC25A13* expression was associated with strong enrichment of MYC targets, E2F targets, oxidative phosphorylation, DNA repair, and G2M checkpoint pathways, highlighting a transcriptional state driven by high biosynthetic demand and replication stress (Figure 9D–F).

Similarly, *SLC38A2* expression correlated with enrichment of MYC signaling, mTORC1 signaling, hypoxia, and cell cycle progression pathways, reflecting adaptive metabolic rewiring that supports proliferation under hypoxic and nutrient-deprived conditions (Figure 9G–I).

Across all analyses, a coherent biological theme emerges: *CP*, *SLC25A13*, and *SLC38A2* converge on transcriptional and signaling programs that integrate glutamine-associated transport and metabolism with inflammation, DNA damage tolerance, cell cycle progression, and stress adaptation. These enrichments represent correlated expression states characteristic of aggressive GBM biology rather than direct evidence of pathway activation or regulatory control by individual genes. Nevertheless, the consistency across GO, KEGG, interaction networks, curated signaling maps, and Hallmark gene sets supports the concept that these three genes occupy central positions within glutamine-driven metabolic and proliferative networks, providing a functional foundation for their prioritization in downstream mechanistic and therapeutic analyses (Figure 7A–I, Figure 8A–C and Figure 9A–I).

### 2.4. Epigenetic Regulation and Immune Microenvironment Associations of CP, SLC25A13, and SLC38A2 in GBM

**Figure 10:**
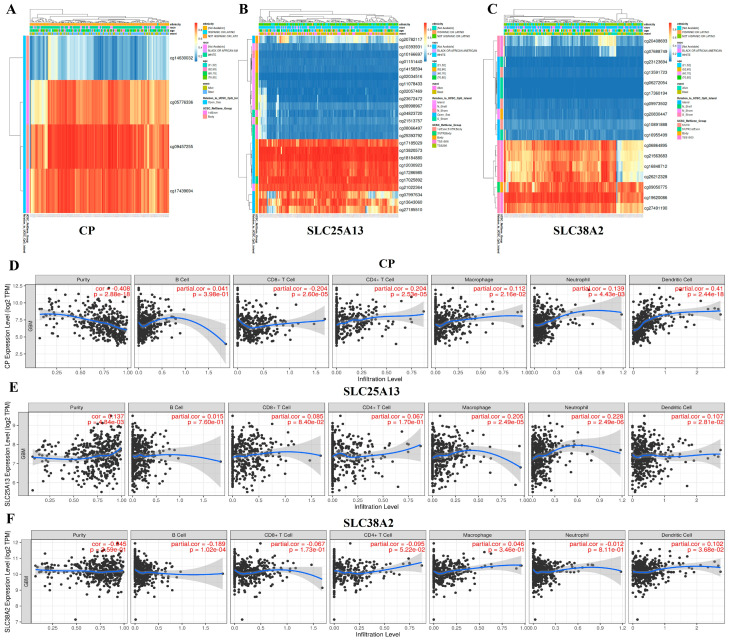
**Epigenetic regulation and immune microenvironment associations of *CP*, *SLC25A13*, and *SLC38A2* in GBM.** (**A**–**C**) Heatmaps depicting DNA methylation profiles of *CP* (**A**), *SLC25A13* (**B**), and *SLC38A2* (**C**) across GBM samples. Rows represent CpG probes mapped to each gene, and columns represent individual tumor samples. Hierarchical clustering was applied to visualize methylation heterogeneity, with color gradients indicating relative methylation levels from hypomethylation to hypermethylation. (**D**–**F**) Correlation analyses between gene expression levels and immune cell infiltration estimates for *CP* (**D**), *SLC25A13* (**E**), and *SLC38A2* (**F**). Scatter plots illustrate associations with tumor purity, B cells, CD8^+^ T cells, CD4^+^ T cells, macrophages, neutrophils, and dendritic cells. Gene expression is shown as log_2_ (TPM), and immune infiltration scores are plotted on the x-axis. Partial correlation coefficients adjusted for tumor purity and corresponding *p*-values are indicated in each panel.

To investigate epigenetic mechanisms underlying the dysregulated expression of *CP*, *SLC25A13*, and *SLC38A2* in GBM, DNA methylation profiles were examined across tumor samples. Gene-centric methylation heatmaps revealed heterogeneous but gene-specific CpG methylation patterns, reflecting distinct epigenetic architectures rather than uniform methylation shifts across the genome (Figure 10A–C). For each gene, CpG probes clustered into hypomethylated and hypermethylated regions, indicating selective epigenetic remodeling in GBM. For *CP*, differential methylation across CpG sites suggests partial promoter and gene-body hypomethylation in a subset of tumors, a configuration frequently associated with transcriptional activation of stress-responsive and metabolic genes (Figure 10A). Such epigenetic relaxation may facilitate sustained CP expression under oxidative and inflammatory conditions characteristic of the GBM microenvironment. *SLC25A13* exhibited pronounced hypomethylation at CpG loci linked to transcriptional regulation, consistent with epigenetic derepression of mitochondrial transport functions required for nucleotide biosynthesis and redox balance (Figure 10B). In contrast, SLC38A2 showed region-specific methylation heterogeneity, suggesting dynamic epigenetic control that may enable adaptive amino acid transport in response to nutrient stress (Figure 10C).

To further explore the biological consequences of these epigenetic and transcriptional states, correlations between gene expression levels and immune cell infiltration were analyzed. Expression of *CP* demonstrated significant associations with tumor purity and multiple immune cell populations, including B cells, CD8^+^ T cells, CD4^+^ T cells, macrophages, neutrophils, and dendritic cells, indicating a close relationship between *CP*-linked metabolic states and immune infiltration patterns (Figure 10D). Given *CP*’s role in iron metabolism and redox regulation, these associations are biologically consistent with known links between iron homeostasis, oxidative stress, and immune cell recruitment. Similarly, *SLC25A13* expression correlated with tumor purity and several immune cell subsets, particularly macrophages, neutrophils, and dendritic cells (Figure 10E). As a mitochondrial aspartate–glutamate carrier, *SLC25A13* supports biosynthetic and proliferative metabolism, which may indirectly shape immune infiltration by altering tumor growth dynamics and metabolic competition within the microenvironment. Expression of *SLC38A2* was also significantly associated with tumor purity and immune infiltration, including CD8^+^ T cells, macrophages, neutrophils, and dendritic cells (Figure 10F). Given its function as an adaptive amino acid transporter upregulated during nutrient deprivation, *SLC38A2*-associated transcriptional programs likely reflect metabolic states that influence immune cell viability and function under hypoxic and nutrient-limited conditions.

Collectively, these findings demonstrate that *CP*, *SLC25A13*, and *SLC38A2* are subject to gene-specific epigenetic regulation and are tightly linked to the immune landscape of GBM (Figure 10A–F). The observed associations reflect coordinated epigenetic, metabolic, and microenvironmental states rather than direct immunoregulatory roles. Nevertheless, the convergence of DNA methylation remodeling, transcriptional activation, and immune infiltration patterns supports the involvement of these genes in shaping the immunometabolic niche of GBM and provides further justification for their prioritization in downstream mechanistic and therapeutic analyses.

### 2.5. Single-Cell Validation of Tumor-Intrinsic Expression of CP, SLC25A13, and SLC38A2

**Figure 11:**
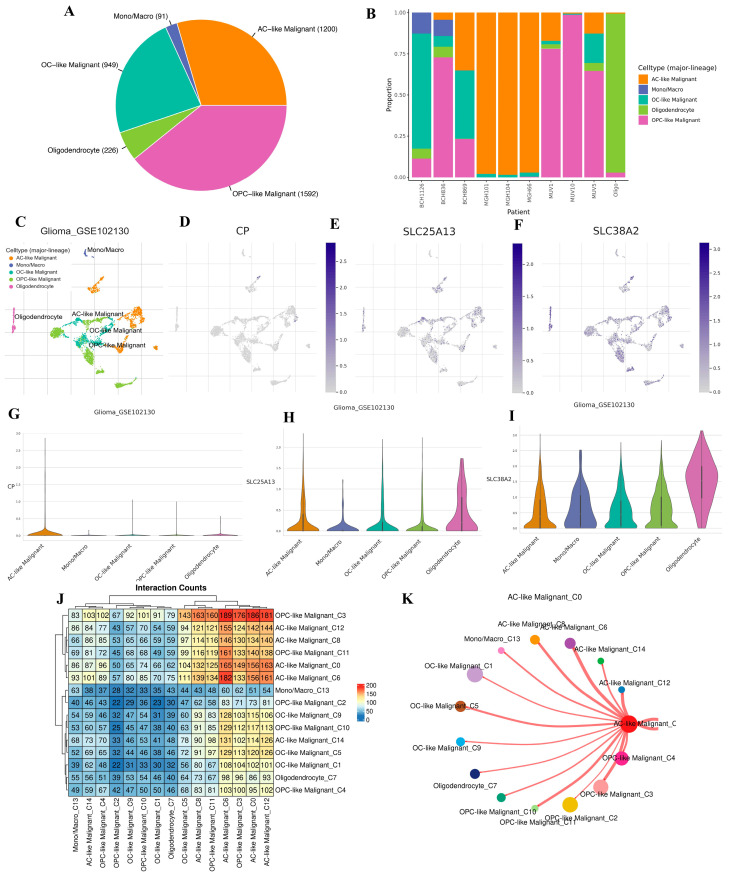
**Single-cell landscape and cellular context of *CP*, *SLC25A13*, and *SLC38A2* expression in GBM using the GSE102130 dataset.** (**A**) Pie chart showing the overall proportion of major cell populations, including AC-like malignant cells, OPC-like malignant cells, OC-like malignant cells, oligodendrocytes, and mono/macrophages. (**B**) Stacked bar plot depicting the relative abundance of major cell lineages across individual patients, highlighting inter-patient heterogeneity in cellular composition. (**C**) UMAP projection of single cells colored by major cell type annotation, illustrating the transcriptional separation of malignant and non-malignant populations. (**D**–**F**) Feature plots overlaid on the UMAP showing the expression patterns of *CP* (**D**), *SLC25A13* (**E**), and *SLC38A2* (**F**) across different cellular compartments. Gene expression intensity is indicated by color gradients. (**G**–**I**) Violin plots summarizing the distribution of *CP* (**G**), *SLC25A13* (**H**), and *SLC38A2* (**I**) expression levels across annotated cell populations, demonstrating cell type–specific expression variability. (**J**) Heatmap showing interaction counts between annotated cell clusters, reflecting the extent of potential intercellular communication among malignant and non-malignant populations. (**K**) Network visualization highlighting interaction patterns centered on AC-like malignant cells, with edge thickness representing interaction strength and node size indicating relative connectivity.

ScRNA-seq analysis was performed to define the cellular landscape and tumor-intrinsic context of *CP*, *SLC25A13*, and *SLC38A2* in GBM. Major cell populations within the tumor ecosystem were first delineated, revealing that malignant cell states dominate the cellular composition, particularly astrocyte-like and oligodendrocyte progenitor–like malignant populations, alongside smaller fractions of non-malignant oligodendrocytes and mono/macrophages (Figure 11A). Substantial inter-patient variability in lineage composition was observed, underscoring the intrinsic heterogeneity of GBM across individuals (Figure 11B). Dimensionality reduction and clustering analysis demonstrated clear transcriptional separation between malignant and non-malignant compartments, confirming robust cell-type annotation and preservation of malignant state identity (Figure 11C). Within this framework, feature plots revealed that *CP*, *SLC25A13*, and *SLC38A2* expression is preferentially enriched within malignant cellular compartments rather than uniformly distributed across all cell types (Figure 11D–F). This localization supports a tumor-intrinsic role for these genes rather than expression driven by immune or stromal cell populations. Violin plot analysis further quantified expression differences across annotated cell populations, revealing cell-type–specific expression patterns (Figure 11G–I). *CP* expression is primarily associated with malignant subclusters linked to stress-adapted transcriptional programs, consistent with its role in iron metabolism and redox regulation. *SLC25A13* expression is elevated in malignant populations characterized by high biosynthetic demand, aligning with its function in mitochondrial aspartate–glutamate exchange and nucleotide biosynthesis. *SLC38A2* exhibits broad expression across malignant states, reflecting its role as an adaptive amino acid transporter supporting metabolic plasticity under nutrient-limited and hypoxic conditions. To assess how these malignant populations interact within the tumor ecosystem, intercellular communication analysis was performed. Interaction heatmaps revealed extensive communication between malignant subclusters and other tumor-associated cell types, indicating that malignant cells act as dominant signaling hubs within the GBM microenvironment (Figure 11J). Network visualization further highlighted astrocyte-like malignant cells as central interaction nodes, exhibiting strong connectivity with multiple malignant and non-malignant populations (Figure 11K). This interaction dominance suggests that malignant cells expressing *CP*, *SLC25A13*, and *SLC38A2* are not only metabolically active but also structurally positioned to influence the broader tumor microenvironment through paracrine and juxtacrine signaling.

Collectively, this single-cell analysis establishes that *CP*, *SLC25A13*, and *SLC38A2* are predominantly expressed within malignant GBM cell states, display cell-type–specific expression heterogeneity, and are embedded within highly interactive malignant communication networks (Figure 11A–K). These findings provide cellular-level validation of bulk transcriptomic results and support a model in which glutamine-associated metabolic genes contribute to GBM progression through tumor-intrinsic metabolic adaptation and coordinated intercellular communication.

### 2.6. Pharmacogenomic Drug Analysis and Structural Validation

**Figure 12:**
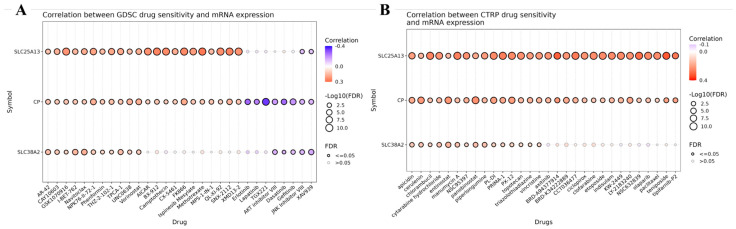
**Association between *CP*, *SLC25A13*, and *SLC38A2* expression and drug sensitivity in cancer cell lines.** (**A**) Bubble plot showing correlations between mRNA expression levels of *CP*, *SLC25A13*, and *SLC38A2* and drug sensitivity profiles derived from the Genomics of Drug Sensitivity in Cancer (GDSC) database. Each bubble represents a drug–gene pair. Color intensity indicates the direction and strength of correlation (blue to violet, negative correlation; red, positive correlation), while bubble size reflects statistical significance expressed as −log10 false discovery rate (FDR). Filled circles denote statistically significant associations (FDR ≤ 0.05), and open circles indicate non-significant correlations. (**B**) Bubble plot illustrating correlations between mRNA expression of CP, SLC25A13, and SLC38A2 and drug sensitivity data from the Cancer Therapeutics Response Portal (CTRP), displayed using the same color and size scales as in panel (**A**).

To translate the pathway-level findings into potential therapeutic vulnerabilities, we systematically evaluated the association between *CP*, *SLC25A13*, and *SLC38A2* expression and drug sensitivity profiles using large-scale pharmacogenomic datasets. Drug–gene correlation analyses were performed using the Genomics of Drug Sensitivity in Cancer (GDSC) and Cancer Therapeutics Response Portal (CTRP) resources, enabling an unbiased assessment of how transcriptional variation in these genes relates to cellular response across diverse cancer cell lines (Figure 12). 

In the GDSC analysis (Figure 12A), several compounds exhibited significant negative correlations between drug response and gene expression, indicating increased drug sensitivity in cell lines with higher *CP*, *SLC25A13*, or *SLC38A2* expression. Importantly, these inverse correlations were observed predominantly for drugs targeting PI3K–AKT–mTOR signaling, mitochondrial metabolism, cell-cycle regulation, and stress-response pathways, which directly aligns with the pathway enrichment patterns identified earlier by Hallmark GSEA and KEGG analyses. In contrast, although some compounds displayed stronger negative correlation magnitudes (darker blue color intensities), these agents were deprioritized when they lacked mechanistic relevance to the metabolic and signaling axes highlighted in our pathway analysis or showed inconsistent significance across datasets.

CTRP-based correlations (Figure 12B) further supported this selection strategy by demonstrating directionally consistent but more moderate associations, reinforcing the robustness of the identified drug–gene relationships while highlighting dataset-specific differences in compound coverage and screening conditions. Together, these analyses emphasize that biological concordance with pathway activation states, rather than correlation strength alone, is critical for rational drug prioritization.

**Figure 13:**
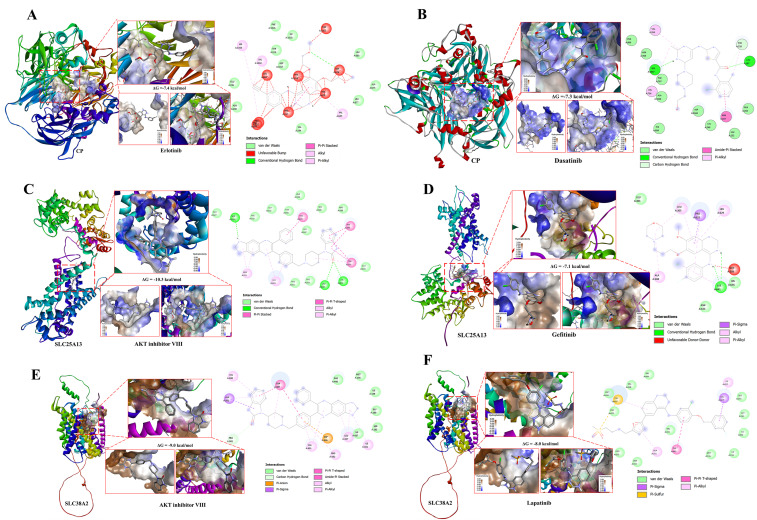
**Molecular docking analysis of BBB-relevant compounds with *CP*, *SLC25A13*, and *SLC38A2*.** (**A**,**B**) Representative three-dimensional docking poses of selected blood–brain barrier (BBB)–penetrant small-molecule compounds within the predicted binding pockets of *CP*, highlighting favorable ligand accommodation within surface cavities and internal grooves. Enlarged views depict the local binding environment and surface electrostatic properties of the interaction sites. Two-dimensional interaction diagrams summarize key non-covalent interactions, including hydrogen bonding, hydrophobic contacts, π–π stacking, and van der Waals forces. (**C**,**D**) Docking conformations of BBB-relevant compounds with *SLC25A13*, illustrating stable ligand positioning within transporter-associated cavities and interaction networks compatible with modulation of mitochondrial metabolite exchange. (**E**,**F**) Docking analysis of BBB-penetrant compounds interacting with *SLC38A2*, demonstrating ligand engagement within putative substrate or regulatory regions of the amino acid transporter. Surface representations and interaction maps highlight hydrogen bonds, π–alkyl interactions, and hydrophobic contacts contributing to binding stability.

Notably, although several compounds exhibited stronger correlation magnitudes in drug sensitivity analyses, they were not automatically prioritized for downstream validation. Drug selection was guided by an integrative framework that extended beyond statistical association to include biological coherence with the identified glutamine-centric metabolic programs, consistency across independent pharmacogenomic datasets, and structural compatibility with the target proteins. Compounds were excluded if they showed discordant behavior between datasets, targeted pathways unrelated to glutamine metabolism, mitochondrial function, or mTOR-linked signaling, or lacked translational feasibility for GBM therapy. Crucially, blood–brain barrier (BBB) permeability was incorporated as a decisive selection criterion, given the anatomical and pharmacological constraints of central nervous system tumors. Only compounds with documented or strongly predicted BBB penetration were advanced, ensuring that selected candidates were not only statistically and biologically relevant but also pharmacologically realistic for brain tumor treatment. Based on these integrated criteria, representative BBB-permeant compounds were subjected to structure-based molecular docking against *CP*, *SLC25A13*, and *SLC38A2* (Figure 13). Structure-based molecular docking was performed to evaluate the binding feasibility and interaction stability of these pathway-aligned, pharmacogenomically selected small-molecule compounds with *CP*, *SLC25A13*, and *SLC38A2* (Figure 13A–F). Docking analyses for *CP* demonstrated stable ligand accommodation within surface-accessible and internal cavities. Erlotinib (Figure 13A) and dasatinib (Figure 13B) exhibited favorable binding orientations supported by hydrogen bonding, hydrophobic contacts, π–π stacking, and van der Waals interactions, with predicted binding free energies of approximately −7.4 kcal/mol and −7.3 kcal/mol, respectively. These interaction profiles are consistent with *CP*’s proposed role in oxidative stress regulation and tumor-associated metabolic adaptation in GBM. For *SLC25A13*, docking revealed stable ligand positioning within transporter-associated cavities relevant to mitochondrial aspartate–glutamate exchange. AKT inhibitor VIII (Figure 13C) displayed strong binding affinity (ΔG ≈ −10.3 kcal/mol), while gefitinib (Figure 13D) showed a favorable interaction energy (ΔG ≈ −7.1 kcal/mol), supported by extensive hydrogen bonding and hydrophobic interactions. These structural features align with pathway analyses implicating *SLC25A13* in anabolic metabolism, nucleotide biosynthesis, and PI3K–AKT–mTOR–associated signaling programs. Docking analyses for *SLC38A2* further demonstrated ligand engagement within putative substrate or regulatory regions of this amino acid transporter. AKT inhibitor VIII (Figure 13E) and lapatinib (Figure 13F) exhibited stable binding conformations with predicted binding free energies of approximately −9.8 kcal/mol and −8.0 kcal/mol, respectively. Interaction maps highlighted hydrogen bonds, π–alkyl interactions, and hydrophobic contacts contributing to binding stability, consistent with the established role of *SLC38A2* in glutamine transport and mTOR-dependent growth signaling.

Across all three targets, the observed docking energies fall within ranges typically associated with biologically meaningful protein–ligand interactions, supporting the structural plausibility of direct target engagement. Importantly, these compounds were prioritized not solely on the basis of correlation strength in drug sensitivity analyses, but through a multilayered selection strategy integrating pathway relevance, pharmacogenomic consistency, BBB permeability, and favorable molecular interaction energetics. Collectively, these findings provide structural validation for *CP*, *SLC25A13*, and *SLC38A2* as therapeutically actionable metabolic nodes in GBM.

## 3. Discussion

GBM is characterized by extreme metabolic plasticity, enabling tumor cells to survive under hypoxic, nutrient-limited, and highly heterogeneous microenvironmental conditions [1]. Among the metabolic adaptations observed in GBM, glutamine utilization has emerged as a critical axis supporting biosynthesis, redox balance, and signaling integration. While prior studies have largely focused on enzymatic components of glutamine metabolism or well-established metabolic drivers such as IDH mutations, comparatively less attention has been given to glutamine-associated transport, exchange, and metabolic coupling nodes that regulate intracellular nutrient flux and metabolic state coordination [6,7,9]. This gap limits our understanding of how GBM selectively rewires glutamine-related programs beyond canonical pathways and restricts the identification of novel, clinically relevant biomarkers.

In this study, we employed an integrated multi-layered analytical framework to systematically dissect glutamine-associated metabolic reprogramming in GBM. Rather than assuming uniform activation of glutamine metabolism, our differential expression analyses demonstrated selective transcriptional deregulation, indicating that GBM preferentially amplifies specific transport and exchange components while suppressing others. This observation highlights an important conceptual distinction: glutamine dependency in GBM is not a global metabolic phenomenon, but rather a modular and context-dependent process driven by a subset of dominant regulatory nodes [10]. Survival analyses further refined this framework by distinguishing genes that are merely differentially expressed from those that are clinically consequential. Although *IDH1* emerged as a significant survival-associated gene, it was intentionally excluded from downstream prioritization due to its extensive characterization and mutation-driven biology in glioma. Instead, we focused on *CP*, *SLC25A13*, and *SLC38A2*, which consistently demonstrated tumor-specific upregulation, adverse prognostic associations, and limited prior characterization in the context of GBM glutamine metabolism. This decision reflects a deliberate emphasis on novelty and translational relevance, rather than rediscovery of established metabolic paradigms [11,12,13].

Functional enrichment, pathway analyses, and protein–protein interaction networks collectively positioned CP, SLC25A13, and SLC38A2 at the intersection of metabolic stress adaptation, biosynthetic demand, and growth-associated signaling programs. CP emerged as a node linked to oxidative stress handling, iron homeostasis, and inflammatory states, all of which are central to GBM survival under hypoxic and oxidative pressure. SLC25A13, as a mitochondrial aspartate-glutamate carrier, connected mitochondrial metabolism to cytosolic nucleotide biosynthesis and redox balance, reinforcing its role in supporting rapid proliferation and DNA damage tolerance. SLC38A2, an adaptive amino acid transporter, was associated with nutrient stress responses and mTOR-linked signaling, underscoring its importance in sustaining glutamine availability in metabolically constrained tumor regions [14,15]. Importantly, protein-level validation and epigenetic analyses reinforced the biological relevance of these findings. The concordance between mRNA upregulation, protein expression, and gene-specific DNA methylation patterns suggests that CP, SLC25A13, and SLC38A2 are actively regulated components of the GBM metabolic state, rather than passive transcriptional byproducts. This multi-layer consistency strengthens their candidacy as robust biomarkers of glutamine-associated metabolic rewiring.

The integration of tumor microenvironment and immune infiltration analyses further contextualized these genes within GBM biology. Rather than implying direct immunoregulatory functions, the observed associations indicate that CP-, SLC25A13-, and SLC38A2-high tumors occupy distinct immunometabolic states, likely reflecting metabolic competition, redox imbalance, and stress signaling that shape immune cell infiltration patterns. These findings align with emerging evidence that tumor metabolism indirectly modulates immune landscapes by altering nutrient availability and metabolic byproducts [16,17]. ScRNA-seq analyses provided critical validation of tumor-intrinsic expression, demonstrating that CP, SLC25A13, and SLC38A2 are preferentially enriched within malignant cell populations rather than stromal or immune compartments. This cellular specificity confirms that the metabolic programs identified in bulk analyses are driven by cancer cells themselves, reinforcing the biological significance of these genes as intrinsic components of GBM metabolic architecture.

Finally, pharmacogenomic correlation analysis and structure-based molecular docking were incorporated as functional validation layers rather than as drug discovery endpoints. Pharmacogenomic analyses using GDSC and CTRP datasets revealed that expression levels of CP, SLC25A13, and SLC38A2 are associated with differential drug sensitivity patterns across cancer cell lines, with negative correlations indicating increased sensitivity in gene-high contexts. However, correlation magnitude alone was not used to prioritize compounds, as several drugs displaying stronger signals were excluded due to inconsistent behavior across datasets, lack of alignment with glutamine-centric pathways identified by GSEA and MetaCore analyses or limited translational relevance. Instead, representative compounds were selected based on concordance across datasets, pathway coherence, and structural feasibility. Specifically, erlotinib and dasatinib were evaluated in relation to CP [18,19,20,21], reflecting their consistent pharmacogenomic associations and compatibility with CP-associated surface and cavity regions implicated in redox regulation and stress adaptation. For SLC25A13, AKT inhibitor VIII and gefitinib were prioritized based on their alignment with PI3K–AKT–mTOR–linked anabolic and nucleotide biosynthesis pathways, as well as their stable docking conformations within transporter-associated regions relevant to mitochondrial metabolite exchange [22,23,24]. Similarly, AKT inhibitor VIII and lapatinib were examined for SLC38A2, consistent with its role in glutamine uptake, nutrient sensing, and mTORC1–MYC–driven growth signaling [25,26]. Docking analyses demonstrated stable ligand accommodation within predicted binding pockets or transporter-associated regions of CP, SLC25A13, and SLC38A2, supported by favorable binding free energies and non-covalent interaction networks, including hydrogen bonding, hydrophobic contacts, and π-interactions. Importantly, these findings were interpreted as evidence of functional plausibility rather than therapeutic efficacy, reinforcing a conservative framework in which CP, SLC25A13, and SLC38A2 are positioned as metabolically actionable nodes warranting future experimental validation rather than immediate clinical translation.

Collectively, this study advances the understanding of glutamine-associated metabolic reprogramming in GBM by shifting the focus from canonical enzymatic pathways to transport- and exchange-centric metabolic control points. CP, SLC25A13, and SLC38A2 emerge as biologically integrated, prognostically relevant, and mechanistically plausible biomarkers that capture key aspects of GBM metabolic adaptation. By bridging transcriptomics, epigenetics, pathway analysis, tumor microenvironment context, single-cell resolution, and pharmacological feasibility, our work provides a comprehensive systems-level framework for identifying and prioritizing metabolic vulnerabilities in GBM. Future studies will be required to experimentally validate the functional roles of these genes and to determine whether their modulation can effectively disrupt glutamine-dependent metabolic states in GBM. Nevertheless, the integrative strategy presented here offers a scalable blueprint for uncovering clinically relevant metabolic biomarkers and highlights the importance of transport and metabolic coupling nodes as underexplored but critical determinants of tumor behavior.

## 4. Materials and Methods

### 4.1. Study Design, Literature Review, and Gene Selection

This study employed an integration in silico framework to investigate glutamine-associated metabolic regulators in GBM using publicly available datasets and open-access bioinformatics platforms. No new biological samples were generated. The analytical workflow comprised literature-guided gene selection followed by transcriptomic profiling, survival analysis, functional enrichment, protein-level validation using immunohistochemistry data from the Human Protein Atlas, protein–protein interaction network analysis, pharmacogenomic association analysis, and structure-based molecular docking.

An initial targeted literature review was conducted using PubMed and Google Scholar to identify glutamine transporters, metabolic enzymes, and regulatory factors previously implicated in cancer biology. Reference was made to a comprehensive glutamine metabolism framework study that systematically curated key components involved in glutamine uptake, utilization, and metabolic regulation. This resource was used solely to inform unbiased gene selection, while all downstream analyses were performed independently in a GBM-specific context. Search terms included glutamine transport, SLC1A5, ASCT2, LAT1, SLC7A5, SLC6A14, glutamine metabolism, GLS, ALDH18A1, and glutamine addiction.

Based on this review, a priori gene set representing core regulators of glutamine metabolism was defined. This set included glutamine transporters (SLC1A5, SLC38A1, SLC38A2, SLC7A11, SLC25A1, SLC25A11, SLC25A12, SLC25A13, and SLC25A22), glutamine metabolism enzymes (GLS1, GLS2, GLUD1, GOT2, and GPT2), enzymes involved in hexosamine and asparagine biosynthesis (GFAT and ASNS), nucleotide synthesis enzymes participating in purine and pyrimidine pathways (PPAT, PFAS, GMPS, CPS1, CPS2, and CTPS), tricarboxylic acid cycle–related metabolic drivers and mutation-associated enzymes (IDH1 and IDH2), and signaling or growth regulators linked to metabolic control (mTORC1, MYC, mtKRAS, and mtLKB).

### 4.2. Differential Gene Expression and Survival Analysis

Differential gene expression analysis between GBM tumor tissue and normal brain tissue was performed using the GEPIA2 platform (http://gepia2.cancer-pku.cn/), which integrates RNA-sequencing data from The Cancer Genome Atlas (TCGA) and the Genotype-Tissue Expression (GTEx) project. Expression values were normalized as log2(TPM + 1), and tumor–normal comparisons were visualized using box plots generated by the GEPIA2 interface. Genes were considered differentially expressed based on a threshold of |log2 fold change| > 1 and a significance level of *p* < 0.05, following the platform’s default statistical pipeline [27].

Overall survival associations for candidate genes were evaluated using the GLIOVIS platform (http://gliovis.bioinfo.cnio.es/), which provides integrated survival analysis across multiple glioma cohorts. Patients were stratified into high- and low-expression groups based on median expression values. Kaplan–Meier survival curves were generated, and statistical significance was assessed using log-rank tests [28,29]. 

### 4.3. Clinicopathological Expression, Protein Expression Validation and Protein–Protein Interaction Network

To examine gene expression patterns across clinicopathological features, including tumor status, age, gender, and race, the UALCAN portal (http://ualcan.path.uab.edu/) was utilized. UALCAN analyzes TCGA RNA-seq data normalized as transcripts per million (TPM). Expression differences were visualized using boxplots with statistical comparisons performed using UALCAN’s default statistical framework [30].

Protein-level expression of selected genes was assessed using immunohistochemistry (IHC) data from the HPA (https://www.proteinatlas.org/). Representative IHC images from normal cerebral cortex and GBM tumor tissues were retrieved to evaluate staining intensity, localization, and cellular distribution [31].

Protein–protein interaction (PPI) networks were constructed using the STRING database (version 11.5; https://string-db.org/). High-confidence interaction networks were generated using a minimum interaction score threshold of 0.7. Both experimentally validated and predicted interactions were included to contextualize candidate genes within metabolic and signaling networks [32].

### 4.4. Pathway and Functional Network Enrichment Analysis

Functional and pathway analyses were performed to identify biological programs associated with gene expression in GBM using R-based workflows and curated pathway databases. Raw RNA-sequencing count data generated using the STAR workflow for the TCGA-GBM cohort were obtained as a *SummarizedExperiment* R package (version ≥1.36.0; Bioconductor, Buffalo, NY, USA). All computational analyses were conducted in R (version 4.4.0). Gene-level raw counts were normalized using the *edgeR* package (version ≥3.44.0), and expression values were subsequently transformed using the *limma*–voom framework (limma, version ≥3.58.0) to model mean-variance relationships inherent to count data. Samples were stratified into gene-high and gene-low groups based on the median expression level of the respective gene calculated from log2-transformed counts per million (logCPM) values. Differential expression analysis between the two groups was conducted using the limma package, and genes were ranked according to moderated t-statistics derived from the fitted linear model.

Functional enrichment analyses were performed using complementary R-based and curated approaches. GO enrichment analysis was conducted using the *clusterProfiler* package (version ≥4.10.0) to evaluate over-represented BP, CC, and MF associated with CP-related transcriptional programs. KEGG pathway enrichment analysis was also performed using *clusterProfiler*, with multiple testing correction applied using the Benjamini–Hochberg false discovery rate (FDR) method.

Gene set enrichment analysis (GSEA) was carried out using the *fgsea* package (version ≥1.28.0) implementing the multilevel algorithm. Ranked gene lists were generated based on limma-derived moderated t-statistics. The Hallmark gene set collection was obtained from the Molecular Signatures Database (MSigDB, version 7.5.1) using the *msigdbr* package (version ≥7.5.1). Enrichment significance was evaluated using permutation-based *p*-values followed by FDR correction, and gene sets with adjusted FDR < 0.05 were considered statistically significant [33].

Genes co-expressed with *CP*, *SLC25A13*, and *SLC38A2* were identified using *cBioPortal* (https://www.cbioportal.org) by selecting the top 10% of genes ranked according to Pearson correlation coefficients within the TCGA-GBM cohort. These correlated gene sets were used to characterize transcriptional programs associated with high expression of each candidate gene. Functional annotation and pathway enrichment analyses were first performed using R-based approaches, followed by curated pathway validation. To complement and refine these findings, pathway enrichment analysis was further conducted using MetaCore™ (version 17.1, Clarivate Analytics, Philadelphia, PA, USA), a curated systems biology platform that integrates experimentally supported molecular interactions and manually annotated signaling networks. MetaCore analysis was used to validate and contextualize enriched pathways identified by GO and KEGG analyses, enabling prioritization of biologically relevant metabolic, signaling, and regulatory processes associated with *CP*, *SLC25A13*, and *SLC38A2* [34].

### 4.5. DNA Methylation and Immune Infiltration Analysis and ScRNA-Seq Validation

DNA methylation profiles of candidate genes were analyzed using *MethSurv* (https://biit.cs.ut.ee/methsurv/, accessed on 25 February 2026), which integrates TCGA DNA methylation array data with corresponding clinical annotations. CpG-level methylation patterns were examined to assess epigenetic regulation and were correlated with gene expression levels to evaluate their potential contribution to transcriptional deregulation in GBM [35].

Tumor immune infiltration analysis was performed using TIMER 2.0 (http://timer.cistrome.org/), which estimates the abundance of immune cell populations using multiple deconvolution algorithms. Associations between gene expression and immune cell infiltration levels were evaluated with adjustment for tumor purity, allowing assessment of immune-metabolic context while minimizing confounding effects of non-tumor cells [36].

ScRNA-seq validation was conducted using the TISCH2 database (https://tisch.compbio.cn/), which provides curated and annotated single-cell transcriptomic datasets for GBM. Cell-type-specific expression patterns of candidate genes were examined using feature plots and violin plots, enabling validation of tumor-intrinsic expression and assessment of expression distribution across malignant, immune, and stromal cell populations. Cell-level expression patterns were interpreted to provide spatial and intercellular context for bulk transcriptomic findings [37].

### 4.6. Drug Sensitivity and Molecular Docking Analysis

Drug–gene correlation analysis was performed using the GSCA platform (https://guolab.wchscu.cn/GSCA/#/, accessed on 26 December 2025). Associations between gene expression and drug sensitivity (IC_50_) were evaluated using data from GDSC and CTRP datasets. Negative correlations indicated increased sensitivity in high-expression contexts. Statistical significance was assessed using FDR-adjusted *p*-values [38].

Protein structures corresponding to selected target genes were retrieved from the RCSB Protein Data Bank (https://www.rcsb.org/) or predicted using AlphaFold (https://alphafold.ebi.ac.uk/) [39], when experimental structures were unavailable. Small-molecule structures were obtained from PubChem (https://pubchem.ncbi.nlm.nih.gov/) [40]. Protein and ligand structures were prepared and optimized according to standard docking protocols prior to molecular docking using AutoDock Vina (version 1.2.3; The Scripps Research Institute, La Jolla, CA, USA) [41]. Docking poses and protein–ligand interaction profiles were visualized using BIOVIA Discovery Studio 2026 (Dassault Systèmes, San Diego, CA, USA), and binding affinity scores were reported as predicted free energies of binding (kcal/mol).

### 4.7. Statistical Analysis

All statistical analyses were performed using R software (version 4.4.0) unless otherwise specified [42]. Continuous variables were summarized using appropriate descriptive statistics, and statistical tests were selected based on data distribution and analytical context. For transcriptomic analyses, gene expression values were log2-transformed where applicable, and differential expression analyses were conducted using established R-based frameworks as described in the corresponding Methods sections. Survival analyses were performed using the Kaplan–Meier method, with statistical significance assessed by the log-rank test. Hazard ratios (HRs) were estimated from Cox proportional hazards models where applicable, and HR values greater than 1 were interpreted as indicating an association with poorer overall survival. Patients were stratified into high- and low-expression groups based on median gene expression values unless otherwise stated. Correlation analyses between gene expression levels and other molecular or clinical variables were performed using Spearman’s rank correlation coefficient, given the non-normal distribution of transcriptomic data. For analyses involving multiple hypothesis testing, FDR correction was applied using the Benjamini–Hochberg method to control type I error. An adjusted FDR < 0.25 was considered statistically significant unless otherwise specified. Functional enrichment analyses, including GO, KEGG pathway enrichment, and GSEA, were evaluated using permutation-based or hypergeometric testing frameworks implemented in the respective software tools. Statistical significance for enrichment analyses was determined based on FDR-adjusted *p*-values, with thresholds defined in the corresponding Methods sections.

All statistical tests were two-sided, and a *p*-value < 0.05 was considered statistically significant unless otherwise indicated. Graphical representations were generated using R-based visualization packages, and all statistical interpretations were made in accordance with the assumptions and limitations of the respective analytical methods.

## 5. Conclusions

**Figure 14:**
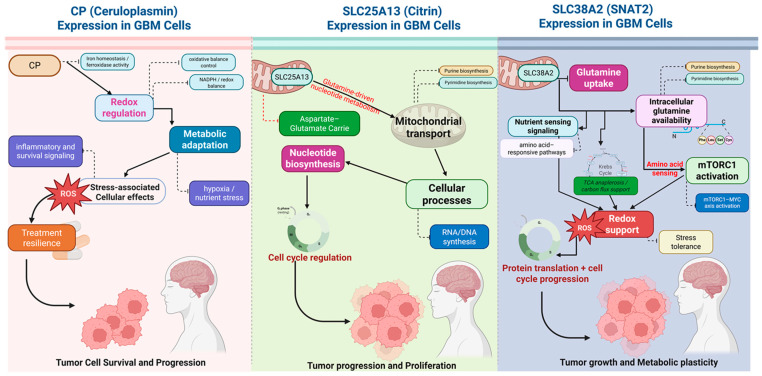
**Schematic overview of *CP-*, *SLC25A13-*, and *SLC38A2*-mediated glutamine-associated metabolic programs in GBM.** The diagram summarizes three complementary metabolic axes identified through integrated transcriptomic, survival, and pathway analyses in GBM. *CP* supports iron homeostasis and redox regulation, limiting oxidative stress and promoting stress-responsive survival signaling and treatment resilience. *SLC25A13* (Citrin) facilitates mitochondrial aspartate–glutamate transport, coupling glutamine-derived metabolites to nucleotide biosynthesis, RNA/DNA synthesis, and cell cycle progression, thereby driving tumor proliferation. *SLC38A2* (*SNAT2*) mediates glutamine uptake and nutrient sensing, leading to mTORC1–MYC axis activation, enhanced protein translation, metabolic stress buffering, and tumor growth. Collectively, these pathways define a coordinated glutamine-associated metabolic network underlying GBM survival, proliferation, and metabolic plasticity.

In conclusion, (Figure 14) this study provides a comprehensive systems-level characterization of glutamine-associated metabolic reprogramming in GBM, with a particular emphasis on transport and metabolic coupling nodes rather than canonical enzymatic pathways. Through integrative analyses spanning transcriptomics, survival stratification, functional enrichment, epigenetic profiling, tumor microenvironment context, and single-cell resolution, we identify *CP*, *SLC25A13*, and *SLC38A2* as biologically coherent and clinically relevant metabolic regulators in GBM. Our findings demonstrate that GBM selectively amplifies specific glutamine-related transport and exchange mechanisms to sustain biosynthetic demand, redox balance, and growth signaling under metabolically constrained conditions. The convergence of tumor-specific expression, adverse prognostic association, coordinated pathway activation, and malignant cell-intrinsic localization underscores the importance of these genes as markers of aggressive metabolic states rather than passive correlates of tumor burden. Importantly, by deliberately excluding well-characterized metabolic drivers such as *IDH1*, this study highlights underexplored but functionally integrated metabolic nodes that may better capture clinically actionable aspects of GBM biology. Beyond descriptive insights, the incorporation of pharmacogenomic correlations and structural interaction analyses provides an additional layer of functional plausibility, supporting the concept that these metabolic nodes are theoretically perturbable. Although not intended as definitive therapeutic validation, this integrative framework establishes a rational foundation for future experimental and translational studies aimed at targeting glutamine-associated vulnerabilities in GBM.

Collectively, this work advances the understanding of GBM metabolism by reframing glutamine dependency as a selective, transport-driven, and context-dependent process. The identification of *CP*, *SLC25A13*, and *SLC38A2* as integrated metabolic biomarkers offers new perspectives on tumor stratification and provides a scalable blueprint for uncovering metabolically actionable targets in highly heterogeneous cancers.

### Limitations

Despite the strengths of this integrative multi-omics approach, several limitations should be acknowledged. First, the majority of analyses in this study are based on publicly available transcriptomic, epigenetic, and pharmacogenomic datasets. While these resources provide substantial statistical power and biological breadth, they inherently reflect population-level trends and may not fully capture patient-specific metabolic heterogeneity or temporal metabolic dynamics during disease progression and treatment. Second, although differential expressions, survival association, and pathway enrichment analyses strongly support the biological relevance of *CP*, *SLC25A13*, and *SLC38A2*, these findings remain correlative in nature. While single-cell analyses and protein-level validation strengthen tumor-intrinsic interpretation, direct functional experiments are required to establish causal relationships between gene modulation and metabolic or proliferative phenotypes in GBM cells. Third, the pharmacogenomic and molecular docking analyses were incorporated as supportive validation layers rather than definitive evidence of therapeutic efficacy. Drug sensitivity correlations derived from cancer cell line panels may not fully recapitulate the metabolic complexity of GBM in vivo, particularly given differences in microenvironmental constraints and blood–brain barrier dynamics. Similarly, molecular docking provides structural feasibility but does not account for dynamic protein conformations, intracellular localization, or pharmacokinetic variability. Fourth, immune infiltration and tumor microenvironment analyses were interpreted conservatively as reflective of immunometabolic states rather than direct immunoregulatory mechanisms. Additional experimental studies, including spatial transcriptomics or metabolic flux analysis in immune–tumor co-culture systems, would be required to delineate causal interactions between glutamine metabolism and immune modulation. Finally, while this study intentionally prioritized underexplored glutamine-associated metabolic nodes to enhance novelty and translational potential, it does not exclude the possibility that additional metabolic regulators outside the selected gene set contribute meaningfully to GBM biology. Future studies integrating metabolomics, isotope tracing, and patient-derived models will be essential to validate and extend the metabolic framework proposed here.

## Data Availability

The original contributions presented in this study are included in the article. Further inquiries can be directed to the corresponding authors.

## Abbreviations

AKT	Protein kinase B
BBB	Blood–brain barrier
BP	Biological process
CGGA	Chinese Glioma Genome Atlas
CP	Ceruloplasmin
CTRP	Cancer Therapeutics Response Portal
DEG	Differentially expressed gene
FDR	False discovery rate
GBM	Glioblastoma multiforme
GDSC	Genomics of Drug Sensitivity in Cancer
GEPIA2	Gene Expression Profiling Interactive Analysis 2
GEO	Gene Expression Omnibus
GO	Gene Ontology
GSEA	Gene set enrichment analysis
GTEx	Genotype-Tissue Expression project
HPA	Human Protein Atlas
IDH	Isocitrate dehydrogenase
KEGG	Kyoto Encyclopedia of Genes and Genomes
logCPM	Log2 counts per million
mRNA	Messenger RNA
mTORC1	Mechanistic target of rapamycin complex 1
MYC	MYC proto-oncogene
NES	Normalized enrichment score
OS	Overall survival
PPI	Protein–protein interaction
RNA-seq	RNA sequencing
ROS	Reactive oxygen species
SLC	Solute carrier
SLC25A13	Solute carrier family 25 member 13 (Citrin)
SLC38A2	Solute carrier family 38 member 2 (SNAT2)
SNAT2	Sodium-coupled neutral amino acid transporter 2
TCGA	The Cancer Genome Atlas
TCA	Tricarboxylic acid cycle
TIMER	Tumor Immune Estimation Resource
TISCH	Tumor Immune Single-Cell Hub
UALCAN	University of Alabama at Birmingham Cancer data analysis portal
